# Identification of QTL controlling domestication-related traits in cowpea (*Vigna unguiculata* L. Walp)

**DOI:** 10.1038/s41598-018-24349-4

**Published:** 2018-04-19

**Authors:** Sassoum Lo, María Muñoz-Amatriaín, Ousmane Boukar, Ira Herniter, Ndiaga Cisse, Yi-Ning Guo, Philip A. Roberts, Shizhong Xu, Christian Fatokun, Timothy J. Close

**Affiliations:** 1 Department of Botany and Plant Sciences, University of California Riverside, Riverside, CA USA; 2 International Institute of Tropical Agriculture, Ibadan, Nigeria; 3 Centre d’Etude Régional pour l’Amélioration de l’Adaptation à la Sècheresse, ISRA/CERAAS, Thies, Senegal; 4 Department of Nematology, University of California Riverside, Riverside, CA USA

## Abstract

Cowpea (*Vigna unguiculata* L. Walp) is a warm-season legume with a genetically diverse gene-pool composed of wild and cultivated forms. Cowpea domestication involved considerable phenotypic changes from the wild progenitor, including reduction of pod shattering, increased organ size, and changes in flowering time. Little is known about the genetic basis underlying these changes. In this study, 215 recombinant inbred lines derived from a cross between a cultivated and a wild cowpea accession were used to evaluate nine domestication-related traits (pod shattering, peduncle length, flower color, days to flowering, 100-seed weight, pod length, leaf length, leaf width and seed number per pod). A high-density genetic map containing 17,739 single nucleotide polymorphisms was constructed and used to identify 16 quantitative trait loci (QTL) for these nine traits. Based on annotations of the cowpea reference genome, genes within these regions are reported. Four regions with clusters of QTL were identified, including one on chromosome 8 related to increased organ size. This study provides new knowledge of the genomic regions controlling domestication-related traits in cowpea as well as candidate genes underlying those QTL. This information can help to exploit wild relatives in cowpea breeding programs.

## Introduction

Plant domestication is considered to be the key technological element of a transition to agriculture^1^. Domesticated crop plants differ from their wild progenitors in various characters collectively termed the “domestication syndrome”^2^. Some of the most important syndrome traits include loss of seed dispersal, early flowering, increased size of the edible organs, and changes in plant architecture and organ coloration^3^, and are the outcome of selection for adaptation to cultivated environments^4^. The genetic control of domestication-related traits (DRTs) has been investigated in numerous crop species, including legumes, mainly by quantitative trait loci (QTL) mapping using crosses between cultivated and wild accessions^5–10^. Increased knowledge of the genomic regions controlling DRTs is valuable to exploit wild germplasm efficiently for improving resistance to biotic and abiotic stresses and overall yield.

Cowpea (*Vigna unguiculata* L. Walp) is one of the main sources of dietary protein and folic acid for millions of people in sub-Saharan Africa. Cowpea is a diploid (2n = 22) with a genetically diverse gene-pool. The wild gene-pool is composed of both perennial and annual types, while cultivated forms are all annual. There are several cultivar groups, of which the most important are subsp. *unguiculata* (grain-type cowpea) and subsp. *sesquipedalis* (yardlong bean)^11^. *V. unguiculata* subsp. *dekindtiana*, a perennial form that only exists in Africa, is considered the wild progenitor of cowpea^12^. However, there is a lack of consensus on where in Africa cowpea domestication occurred. Several domestication locales have been proposed including West and Southeast Africa^13–17^.

Domestication of cowpea has, in general, resulted in a determinate growth habit, increased pod and seed size, early flowering, and reduction of pod shattering. Cultivated cowpea also shows a wide range of flower and seed coat colors, whereas wild cowpeas typically have purple flowers and dark mottled seed coats. Only a few studies have identified QTL controlling DRTs in cowpea. Andargie *et al*.^18^ reported QTL for six DRTs including pod shattering, days to flowering, ovule number and growth habit. In subsp. *sesquipedalis*, QTL for DRTs including pod shattering, pod length and flower color have been also reported^19–22^. However, to date no specific gene underlying any domestication trait has been reported in cowpea.

Currently available genomic resources, which include an annotated reference genome (www.phytozome.net) and a SNP genotyping platform^23^, have enabled new insights into the genetics of domestication of cowpea. In the present study, we investigated the genetic control of nine DRTs in cowpea including pod shattering, seed size, and flowering time using a population of 215 recombinant inbred lines (RILs) derived from a cross between a cultivated and a wild cowpea. The Cowpea iSelect Consortium Array, including 51,128 single nucleotide polymorphism (SNPs) markers^23^, was used to genotype this population, which enabled high density genetic mapping. Using the annotated reference genome, we report genes within QTL regions that control DRTs. This study provides new insights into the genetic control of domestication-related traits in cowpea and can help to utilize more efficiently wild germplasm in breeding programs.

## Results

### Phenotypic variation in the population

Nine traits (pod shattering, peduncle length, flower color, days to flowering, seed weight, pod length, leaf length, leaf width, and seed number per pod) related to domestication in cowpea that differed between the parents were evaluated in the population. The mean of 100-seed weight, seed number per pod, primary leaf length and width, and pod length of the cultivated parent (IT99K-573-1-1) were all higher than those of the wild parent (TVNu-1158), whereas the mean of peduncle length and days to flowering were higher in the wild parent than the cultivated parent (Table 1). The segregation pattern of pod shattering fit the expected Mendelian ratio of 1:1 (P value = 0.25), while significant segregation distortion was observed for flower color (P value = 0.0035). The frequency distribution of the seven remaining traits are shown in Supplementary Fig. S1. Transgressive segregation was observed for peduncle length, days to flowering, seed number per pod, primary leaf length, and pod length (Table 1).

**Table 1:** Mean, standard error, range, and skewness values for the parental lines and the F8 RIL population.

Trait	IT99K-573-1-1	TVNu-1158	RIL population		
			*Mean* +/− *SE*	*Range*	*Skewness*
Pod shattering	0	1	—	—	—
Peduncle length (cm)	15.70 +/− 0.98	20.73 +/− 1.33	16.76 +/− 0.41	5.54–33.8	0.70
Flower color	0	1	—	—	—
Days to flowering	41.00 ± 1.00	70.00 +/− 2.00	47 +/− 1	35–88	1.85
100-seed weight (g)	19.6 +/− 0.36	0.88 +/− 0.01	5.07 +/− 0.17	1.77–11.89	0.76
Pod length (cm)	17.89 +/− 1.13	6.82 +/− 0.36	16.75 +/− 0.40	5.07–20.9	0.88
Leaf length (cm)	7.2 +/− 0.33	2.76 +/− 0.12	4.85 +/− 0.08	2.8–8	0.23
Leaf width (cm)	3.94 +/− 0.26	1.39 +/− 0.11	2.27 +/− 0.05	1–4	0.65
Number of seeds/pod	11 +/− 1	9 +/− 1	9 +/− 1	2–16	0.06

### Development of the cultivated x wild genetic map

A total of 17,739 polymorphic SNPs and 170 RILs which passed quality controls (see Materials and Methods) were used for genetic map construction. All SNPs were mapped into 1,825 bins on 12 linkage groups (LGs). LGs were named and oriented based on cowpea pseudomolecules (www.phytozome.net), from Vu01 to Vu11. Note that this numbering of LGs differs from the one used in previous cowpea genetic maps^23–25^. A cross reference to the previous nomenclature is included in Table 2. One chromosome (Vu03) was separated into two LGs, which were joined based on the consensus genetic map of Muñoz-Amatriaín *et al*.^23^. Based on the consensus map, the gap was estimated at 18 cM and was attributed to the absence of polymorphisms between the parents and to the presence of highly distorted markers. The genetic map covered 1,026.03 cM, with an average density of one marker bin per 1.8 cM, and 9.72 SNPs per bin. The length of the LGs varied from 15.25 (Vu04) to 139.72 cM (Vu03) (Table 2). This genetic map contains the highest number of SNPs in an individual cowpea map to date. Compared to the previously published genetic maps of cowpea constructed with SNP markers^23–25^, we observed a much shorter Vu04 (old LG11). The difference may be due to genomic divergences between the cultivated and wild parents leading to disturbed chromosome pairing which reduces or suppresses recombination in affected regions, and/or structural chromosomal rearrangement. The IT99K-573-1-1 × TVNu-1158 genetic map can be found in Supplementary Table S1.

**Table 2:** Number of SNPs and length of each linkage group in the cultivated x wild cowpea genetic map.

Linkage Group (LG, old)	Chromosome	SNPs	Bins	cM
4	Vu01	1742	132	65.31
7	Vu02	1368	140	98.62
3	Vu03	2780	280	157.61
11	Vu04	117	24	15.25
1	Vu05	1740	205	114.62
6	Vu06	1676	150	78.29
2	Vu07	1968	228	134.15
5	Vu08	1607	159	81.59
8	Vu09	1774	189	100.37
10	Vu10	1416	144	77.37
9	Vu11	1551	174	102.85
**Total**	**Total**	**17739**	**1825**	**1026.03**

### Identification of domestication-related QTL and candidate genes

**Figure 1:**
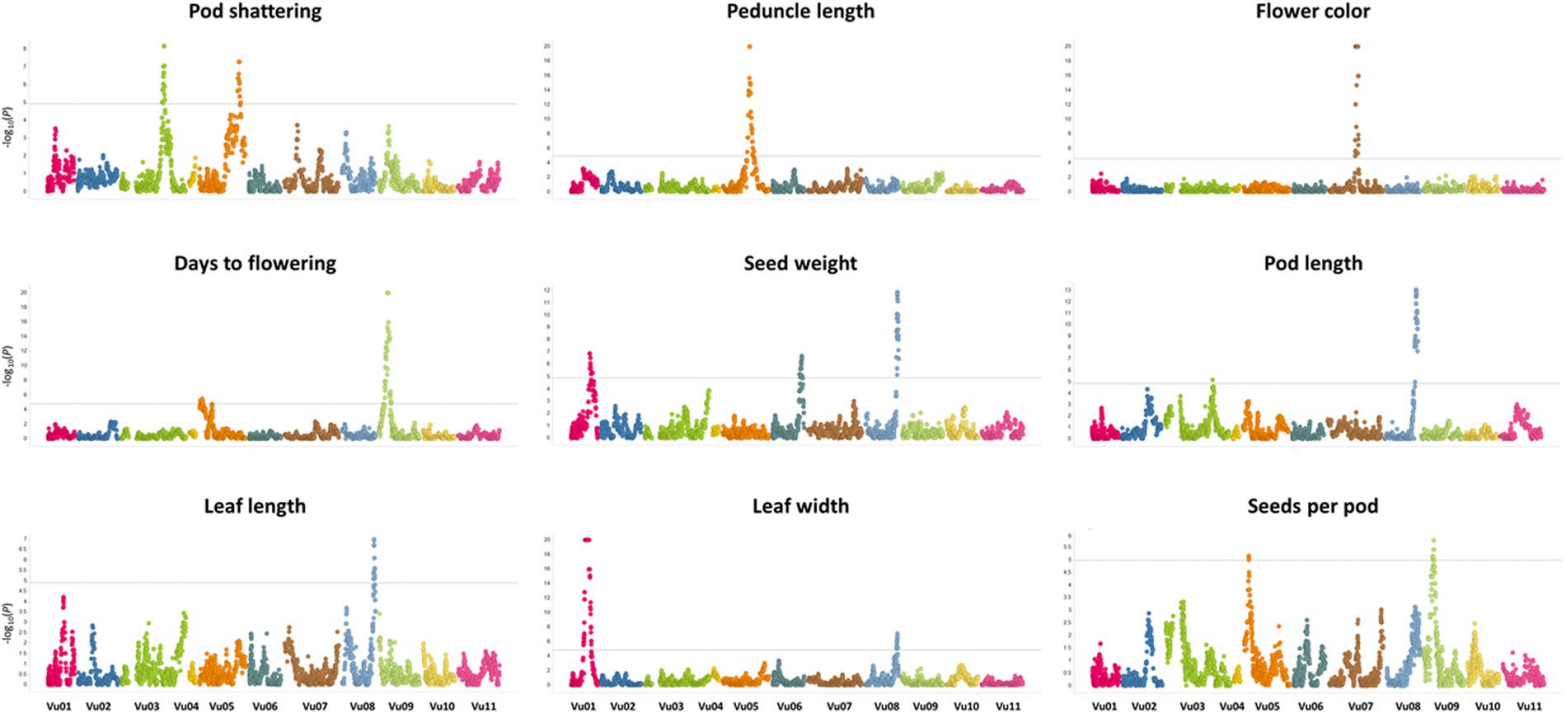
QTL plots for the nine domestication-related traits. The horizontal axis indicate the chromosomes, the vertical axis indicates the −log_10_ of the probability (*P*-values). The dashed line indicates the significance threshold at 0.05.

A total of 16 QTL were identified for the nine traits using a mixed model for QTL mapping^26^ implemented in R. The 16 domestication-related QTL were distributed on seven of the eleven chromosomes of cowpea (Fig. 1; Table 3) and their −log_10_ (*P*-value) ranged from 5.15 for seed number per pod to 20.00 for peduncle length, flower color, and leaf width (Table 3). The significance levels ranged from 4.57 to 4.98 depending on the trait (Supplementary Table S2). The percentage of phenotypic variation for the identified QTL ranged from 18.32% for seed number per pod to 85.65% for flower color (Table 3). We identified four regions showing QTL clustering for domestication traits, including one region on Vu08 where four QTL related to increased organ size (seed weight, pod length, leaf length and leaf width) were mapped. Further studies would be required to determine if this clustering of domestication-related QTL results from pleiotropic effects or tightly linked QTL.

**Table 3:** QTL for the domestication-related traits identified by the linear mixed model analysis and their map position.

Trait	QTL^a^	Peak SNP	Chr Vu	Position (cM)	−Log10(P)	QTL region (cM)	% Phenotypic variation	Effect^b^
Pod shattering	*CPshat3*	2_45094	03	104.84	8.17	100.57–106.67	37.69	−0.31
	*CPshat5*	2_23044	05	100.02	7.28	96.11–103.85	30.27	−0.28
Peduncle length	*CPedl5*	2_00251	05	66.23	20	60.97–76.34	71.83	−4.50
Flower color	*CFcol7*	2_24259	07	65.89	20	64.39–72.43	85.65	−4.16
Days to flowering	*CFt5*	2_05332	05	5.77	5.51	0.93–7.93	20.00	4.13
	*CFt9*	2_03945	09	21.79	20	13.82–30.02	79.30	−8.46
100-seeds weight	*CSw1*	2_17042	01	46.62	6.85	45.15–49.45	19.85	1.03
	*CSw6*	2_55420	06	72.16	6.66	69.26–73.30	21.48	1.06
	*CSw8*	2_05809	08	78.57	11.86	76.14–81.59	36.87	1.39
Pod length	*CPodl3*	2_49508	03	116.49	5.19	116.02–116.49	23.98	1.08
	*CPodl8*	2_00195	08	78.17	13.04	74.67–81.59	46.08	1.52
Leaf length	*CLl8*	2_09764	08	78.87	6.98	78.17–80.02	38.24	0.69
Leaf width	*CLw1*	2_19941	01	34.13	20	30.35–49.45	63.28	0.54
	*CLw8*	2_21200	08	78.87	7.09	76.73–79.18	34.75	0.40
N° seed per pod	*CSp5*	2_07573	05	11.37	5.15	10.57–12.11	18.32	1.07
	*CSp9*	2_19332	09	24.73	5.77	22.51–25.32	21.09	−1.18

^a^QTL name is designated as follow: “C” to indicate cowpea, followed by the trait code, then followed by the chromosome number.

^b^Positive or negative effect alleles, for which a positive value indicates allele of the cultivated parent is positive, and a negative value indicates the allele of the wild parent is positive.

For all the traits and QTL below, additional information on SNP positions within the pseudomolecules, including distance to genes, is available through phytozome (www.phytozome.net).

#### Pod shattering

Two significant QTL were detected for pod shattering, one each on Vu03 and Vu05 (Fig. 1; Table 3). These QTL, named *CPshat3* and *CPshat5*, explained 37.69% and 30.27% of the phenotypic variation, respectively. *CPshat3* spanned 6.1 cM corresponding to ~3.4 Mb on the cowpea pseudomolecules (www.phytozome.net) and contained 268 genes, while *CPshat5* spanned 7.74 cM corresponding to ~1.60 Mb and contained 204 genes (Supplementary Table S3). Among the genes underlying the main QTL (*CPshat3*) two encoding transcription factors stand out in the context of pod shattering, *Vigun03g306000* and *Vigun03g302600* (see Discussion). Among the annotated genes underlying the *CPshat5* region, *Vigun05g273500*, annotated as Myb domain protein 26, is considered to be an especially interesting candidate (see Discussion).

#### Peduncle length

QTL analysis detected one major QTL on Vu05, *CPedl5*. This QTL accounted for 71.83% of the phenotypic variation and it spanned a 15.37 cM region corresponding to ~4.53 Mb (Fig. 1; Table 3). A total of 379 annotated genes were identified in the *CPedl5* interval (Supplementary Table S3). Notably, three genes encode auxin-responsive GH3 family proteins (*Vigun05g201700*, *Vigun05g217600* and *Vigun05g223100*) were located in the interval, and members of the GH3 family have been found to influence organ elongation^27,28^ (see Discussion).

#### Flower pigmentation

A major-effect QTL controlling purple flower (*CFcol7*) was detected in a 64 cM region (~4.56 Mb) on Vu07 (Fig. 1; Table 3). This QTL explained 85.65% of the phenotypic variation and contained 254 annotated genes (Supplementary Table S3). The transcription factor TRANSPARENT TESTA8 (TT8, *Vigun07g110700*), which contains a basic helix-loop-helix domain, was identified in this QTL region (see Discussion).

#### Days to flowering

Two significant QTL related to days to flowering were detected, one each on Vu05 and Vu09. The QTL on Vu05 (*CFt5*) explained 20% of the phenotypic variation, while *CFt9* on Vu09 explained 79.3% of the phenotypic variation (Fig. 1; Table 3). *CFt5* spans 7 cM which correspond to ~6.64 Kb, while *CFt9* maps to a 16 cM region corresponding to ~3.86 Mb. A total of 86 genes were identified in the *CFt5* region, while 299 genes were identified underlying *CFt9* (Supplementary Table S3). Among the annotated genes in the *CFt9* region, a phytochrome E photoreceptor (*Vigun09g050600*) and a transcription factor TCP 18 (*Vigun09g062200*) were found. No genes previously shown to have a role in flowering were identified in the *CFt5* region (see Discussion).

#### 100-seed weight

Three QTL for seed weight were detected, one each on Vu01, Vu06 and Vu08. The QTL with the highest contribution to the trait (*CSw8*) was located on Vu08 and explained 36.87% of the phenotypic variation, while *CSw1* and *CSw6* explained 19.85% and 21.48% of the phenotypic variation, respectively (Table 3). *Csw8* spans 5.45 cM (~1.61 Mb) and contains 225 genes, while *Csw1* and *Csw6* span 4.3 cM (~1.60 Mb) and 4.04 cM (~1.12 Mb) and contain 206 and 160 annotated genes, respectively (Supplementary Table S3). Among the many genes in these QTL regions, several were involved in carbohydrate metabolism, including UDP-glycosyltransferases and cellulose synthases were identified (Supplementary Table S3).

#### Pod length

Pod length was analyzed as a measure of increase in organ size, and two QTL were identified, one each on Vu03 and Vu08. These QTL explained 23.98% and 46.08% of the phenotypic variation and spanned 0.47 cM (~84 Kb) and 6.92 cM (~2.1 Mb), respectively. Nine genes including an AGAMOUS-like8 gene (*Vigun03g343500*), which is known to mediate cell differentiation during fruit development in Arabidopsis^29^, were identified underlying *CPodl3*, while 309 genes were found in the *CPodl8* interval. *CPodl8* maps to the same genomic region as *CSw8* (Fig. 1; Table 3) and includes genes encoding cellulose synthases, UDP-glycosyltransferases and a cluster of pectin lyases (Supplementary Table S3), all involved in carbohydrate metabolism (see Discussion).

#### Leaf length and width

The leaf is the main organ for photosynthesis in cowpea and one QTL for leaf length (*CLl8*) and two QTL for leaf width (*CLw1*, *CLw8*) were identified in this population (Fig. 1; Table 3). *CLl8* explained 38.24% of the phenotypic variation and spanned 1.85 cM (~9.9 Kb). *CLw1* accounted for 63.28% of the phenotypic variation and spanned 19.1 cM (~3.61 Mb), while *CLw8* explained 34.75% of the phenotypic variation and spanned 2.45 cM (~1.08 Mb). Regarding leaf length, a total of 142 genes were identified in the physical region of *CLl8*. For leaf width, over 300 genes were identified in *CLw1* interval, while 148 genes were identified underlying *CLw8* (Supplementary Table S3). *CLl8* and *CLw8* map to the same genomic region as *CSw8* and *CPodl8* (Fig. 1; Table 3). Genes present in *Csw8* and *CPodl8* are also contained in these two QTL, including proteins of the pectin lyase-like superfamily and cellulose synthases (Supplementary Table S3 and Discussion).

#### Seed number per pod

Two QTL were detected, one each on Vu05 and Vu09, accounting for 18.32% and 21.09% of the phenotypic variation, respectively, spanned 1.54 cM (∼3.71 Kb) and 2.81 cM (~4.94 Kb), respectively. The total number of genes underlying *CSp5* and *CSp9* was only 40 and 33, respectively. One of the genes underlying *CSp9* encodes a transcription factor TCP5, which has been associated with ovule development in Arabidopsis^30^. No gene was identified in the *CSp5* region with known functions related to seed number per pod (Supplementary Table S3 and Discussion).

## Discussion

Through the domestication process, cowpea underwent many phenotypic changes compared to its wild progenitor. In this study, we have focused on nine traits that differ between wild and cultivated cowpea and identified 16 QTL for trait determination distributed over seven chromosomes. For each trait, only one or two major QTL were identified except for seed weight, for which three QTL were detected. These results are consistent with previous studies suggesting that, in most crops, domestication related traits seem to be controlled by a small number of QTL with large phenotypic effects^31,32^.

Among the domestication traits considered in this study, loss of pod shattering and time to flowering are the most relevant for cowpea breeding, together with increase of organ size. The pod shattering habit causes pre-harvest yield losses. Two major QTL were identified for pod shattering, one each on Vu03 and Vu05. In previous studies in cowpea, Suanum *et al*.^20^ reported one major QTL and one minor QTL for pod shattering, while Andargie *et al*.^18^ identified only one QTL explaining 10.8% of the phenotypic variation. While it was not possible to compare the QTL identified by Suanum *et al*.^20^ because of unavailability of marker sequences, BLAST searches of SSR markers from the Andargie *et al*.^18^ study to the reference genome sequence (www.phytozome.net) revealed that the QTL identified in that study is located in a different chromosome compared to those reported here. This suggests the QTL identified in this study are novel.

Genes involved in seed dispersal have been cloned in dicots including soybean^33^, Brassica^34^ and Arabidopsis^35^. For *CPshat3*, the major pod shattering QTL, we identified a gene *Vigun03g306000*, which encodes a NAC domain transcription factor (NAC007) involved in secondary cell wall biosynthesis^36^. In soybean, the NAC family gene *SHATTERING1-5* has been found to activate secondary wall biosynthesis affecting pod shattering resistance^35^. In addition, a C2H2 zinc finger protein (*Vigun03g302600*) was identified in this QTL region. A member of the C2H2 zinc finger family of proteins has been shown to enhance silique shattering resistance in Brassica^34^. In the *CPshat5* region we identified *Vigun05g273500*, a gene annotated as Myb domain protein 26. *Vigun05g273500* is an orthologue of *AT3G13890.1*, which acts upstream of the lignin biosynthesis pathway and has been shown to be key for anther dehiscence by regulating the development of secondary thickening in the endothecium in Arabidopsis^37^.

Time to flowering is one of the most important agronomic traits that plays a key role in the adaptation of a variety to specific agro-ecological areas. Two major QTL associated with days to flowering have been identified in this work. As with pod shattering, none of these QTL seem to coincide with the main QTL reported by Andargie *et al*.^18^. The main QTL for days to flowering (*CFt9*) in the present study mapped to Vu09, with the wild accession alleles conferring late flowering. There are two genes in the *CFt9* region with functions related to time to flowering. One is a phytochrome E (*Vigun09g050600*), one of the photoreceptors perciving red/far-red light ratio and influencing flowering time^38^. *Vigun09g050600* is an ortholog of the adzuki bean *Vigan.02G285600.01*, which is one of the candidate genes for the major photoperiod QTL *Flowering Date1*^39^. The other gene is *Vigun09g062200*, encoding a transcription factor TCP 18 which has been shown in Arabidopsis to repress flowering by interacting with the florigen proteins FLOWRING LOCUS T (FT) and TWIN SISTER OF FT (TSF)^40^.

Larger seeds play a major role in consumer preference, and larger leaves provide more surface than smaller leaves for the production of photosynthate. Four traits including 100-seed weight, pod length, primary leaf length and width were analyzed as a measure of increased organ size in the population. QTL identified for seed weight (*CSw8*), leaf length (*CLl8*), leaf width (*CLw8*), and pod length (*CPodl8*) mapped to the same region on Vu08. Multiple QTL located in this region suggest potential pleiotropy or clustering of genes controlling increased organ size, a fundamental target during domestication. In rice bean, QTL for leaf size were detected close to QTL controlling seed- and pod-size related traits^41^. A cluster of pectin lyase-like superfamily proteins, which are known to be involved in many biological processes including cellular metabolism^42^, was found in the QTL region for 100-seed weight, pod length and leaf size. Also, clusters of other genes with functions in carbohydrate metabolism such as UDP-glycosyltransferases and cellulose synthases were identified in this region. Further work, including fine mapping of this hotspot region, would be needed to elucidate the genetic control of increased organ size in cowpea.

Two additional QTL related to seed weight were also detected outside this region, one each on Vu01 and Vu06. One of these (*CSw6*) mapped to the same region as C*ss-4*, a seed size QTL identified from a cultivated by cultivated RIL population^43^. Two other previous studies on cowpea reported QTL involved in seed weight using wild x cultivated population^18,44^. While it was not possible to compare QTL regions between this study and that of Fatokun *et al*.^44^ because of unavailability of marker sequences, none of these QTL seem to coincide with the ones reported by Andargie *et al*.^18^.

As one of the major yield components, the number of seeds per pod is an important trait in cowpea breeding. Two QTL, *CSp5* and *CSp9*, were detected for this trait on Vu05 and Vu09, respectively. The main QTL (*CSp9*) accounted for 21.09% of the phenotypic variation and co-localized with the main QTL for days to flowering. The allele from the wild parent at *CSp9* conferred a higher number of seeds per pod, making it a good target for introgression into cultivated cowpea varieties. *Vigun09g060700* was identified in *Csp9* region. This gene is annotated as a transcription factor TCP5, which is involved in ovule development in Arabidopsis^30^. Since the number of seeds is determined among other things by the number of ovules (and fertile ovules) per ovary, this gene is a promising candidate for number of seeds per pod in cowpea.

Another major difference observed between the wild and the cultivated parent used in this study was the length of the peduncles. A single QTL with a high contribution to the phenotypic variation of the trait (71.83%) was identified on Vu05. Long peduncles in cowpea are desirable as they allow pods to be positioned above the canopy, a characteristic that reduces damage to pods by the pod borer *Maruca vitrata* and are also advantageous for harvesting of pods. Some genes involved in plant growth and development were found underlying *CPedl5*, including genes belonging to the auxin-responsive GH3 family. In cucumber, two genes belonging to this family (*Csa6G492310* and *Csa6G493310*) were identified as candidates for the main QTL for fruit peduncle length^45^.

The presence or absence of purple pigment in the flower is controlled by a single major QTL in this population. This is similar to a previous study in soybean, where one QTL for flower color was identified^46^. The QTL controlling flower color was mapped to Vu07 and explained 85.65% of the phenotypic variation for the trait. An earlier study of cowpea suggested that a single gene controls flower color with purple flower color being dominant^47^. *Vigun07g110700*, involved in flavonoid biosynthesis, was identified in the QTL region. *Vigun07g110700* has sequence similarity to the Arabidopsis *TT8* gene (*AT4G09820.1*) encoding a basic helix-loop-helix domain protein involved in the control of flavonoid biosynthesis, whose final compounds include anthocyanins^48^. *Medicago truncatula TT8* (*MtTT8*) has also been found to regulate anthocyanin and proanthocyanidin biosynthesis by interacting with other transcription factors and forming regulatory complexes^49^. Hence, *Vigun07g110700* is a strong candidate for the control of flower pigmentation in cowpea.

In summary, this study provides novel QTL for many DRTs as well as a link between genetic and physical maps. Thanks to the new genomic resources available for cowpea, which include a reference genome sequence of IT97-499-35 (Lonardi *et al*. in preparation; available from www.phytozome.net), we were able to estimate physical sizes for all QTL identified and to determine, for the first time in cowpea, candidate genes underlying QTL controlling DRTs. The results of this study provide a basis for further fine mapping of genes involved in cowpea domestication and a genetic foundation for the utilization and exploitation of wild relatives in cowpea breeding programs.

## Materials and Methods

### Plant material and growth conditions

A biparental mapping population of 215 F8 RILs derived from a cross between IT99K-573-1-1 and TVNu-1158, cultivated and wild cowpea accessions, respectively, was used for linkage mapping and QTL analysis. IT99K-573-1-1 is an early-maturing, white-seeded, high yielding and Striga resistant variety that was released in Nigeria under the name SAMPEA 14. TVNu-1158 is small seeded and has a perennial growth habit. It is cross-compatible with cultivated cowpea, although the F1 and subsequent generations showed partial sterility.

The parents and the F8 population were grown in pots filled with 5.0 kg topsoil and placed in a screen house at the International Institute of Tropical Agriculture (IITA), Ibadan, Nigeria (latitude 7°30′N and longitude 3°54′E, elevation 240 masl). Five seeds of each RIL were sown per pot and then thinned to two plant per pot when they were well established. The pots were watered regularly.

### Phenotypic data collection and analyses

A total of nine DRTs (pod shattering, peduncle length, flower color, days to flowering, seed weight, pod length, leaf length, leaf width, and seed number per pod) segregating in the population were evaluated. Phenotypic data for pod shattering were collected by visual scoring, with scores of 0 = “no shattering”, and 1 = “pods opened and twisted”. Peduncle length was determined by measuring the distance from the point of peduncle attachment to the node on the stem to where the first flower bud emerged. Five peduncles were measured on each plant beginning with the lowest peduncle. Flower color was evaluated by visual scoring with 0 = “white”, and 1 = “purple”. Flowering time was scored as the number of days from date of planting to when the first flower opened. Seed weight was determined by the weight in grams of 100 seeds. The length (from base to the tip) and width (at the widest point) of the first leaf (opposite in arrangement) following seedling emergence was determined using a measuring tape. Pod length was determined by measuring the length of the first five pods per plant. Seed number per pod was evaluated by counting the number of seeds per 10 pods for each plant.

For the seven non-binary traits, the mean, standard error, and range were calculated as well as the degrees of skewness. The frequency distribution of the seven traits was determined. The segregation pattern for pod shattering and flower color was analyzed by chi-square test for goodness-of-fit.

### Genotyping

Young leaves from each RIL and the parents were collected and placed into a ziplock bag containing silica gel packs for desiccation. Total genomic DNA of each line and parents was extracted from dried leaves using Plant DNeasy (Qiagen, Germany), quantified using Quant-IT dsDNA Assay Kit (Thermo Fisher Scientific, USA), and the concentration adjusted to 80 ng/µl. Genotyping was performed at the University of Southern California (Los Angeles, CA, USA) using the Cowpea iSelect Consortium Array, which includes 51,128 SNPs^23^.

### Linkage map construction

SNPs were called using the GenomeStudio software V.2011.1 (Illumina, Inc., San Diego, CA, USA). Data curation was performed by removing SNPs with more than 20% missing or heterozygous calls. Individuals were then plotted by missing calls and by heterozygous calls, and outliers were eliminated from further analysis. Lines carrying non-parental alleles and duplicated lines were also removed. Only SNPs that were polymorphic in both the parents and the RIL population, and had minor allele frequencies (MAFs) >0.25 were used for linkage mapping. MSTmap^50^ (http://www.mstmap.org/) was used for genetic map construction, with the following parameters: grouping LOD criteria = 10; population type = DH (doubled haploid); no mapping size threshold = 2; no mapping distance threshold: 10 cM; try to detect genotyping errors = no; and genetic mapping function = kosambi. The chromosomes were numbered and oriented according to the recently developed cowpea pseudomolecules (Lonardi *et al*. in preparation; www.phytozome.net). Since the use of DH function inflated the cM distance for a RIL population, cM map distances were divided by two to correct for the extra round of effective recombination occurring in a RIL population compared to a DH population.

### QTL analysis and identification of candidate genes

QTL analysis was performed using a linear mixed model implemented in R and described by Xu^26^. In this approach, the background effect is captured via a polygene and a marker inferred kinship matrix^26,51^. To reserve a window around the test marker, markers around a ±2 cM window at the tested marker were excluded from the kinship matrix. The effect of each marker was estimated as a fixed effect and tested using the Wald test statistic (squared effect divided by the variance of the estimated effect). Under the null model, the Wald test follows a chi-square distribution with one degree of freedom, from which a p-value was calculated for each marker. SNP markers of the entire genome were scanned and a test statistic, −log_10_ (*P*-value), profile was plotted. A genome-wide critical value was calculated with a modified Bonferroni correction using a trait specific “effective number of markers or effective degrees of freedom” as the denominator^52^. The effective degrees of freedom was defined as

$$m0=\frac{{\sum }^{}(Wk-1)}{Wk}$$

where Wk is the Wald test statistic for SNP k. The trait specific Bonferroni corrected critical value was −log_10_ (0.05/m0). A SNP was declared as significant if its −log_10_ (*P*-value) was larger than −log_10_ (0.05/m0). For each significant SNP, an estimate of the percentage of phenotypic variation was calculated. The proportion of phenotypic variance contributed by each SNP was calculated using the following formula:

$$\frac{var\,(X){a}^{2}}{var\,(y)}$$

where X is a variable holding the genotype code (+1, −1) for SNP k, var(X) is the variance of variable X, a is the estimated effect for SNP k and var(y) is the total phenotypic variance of the trait under study.

The physical region of each QTL was determined from the cowpea genome V.1.0 (Lonardi *et al*. in preparation; www.phytozome.net), and used to identify candidate genes underlying the QTL interval. Gene models along with their functional annotation were obtained from the Joint Genome Institute cowpea genome portal (www.phytozome.net).

### Data availability statement

The data analyzed in this study are included in the submitted manuscript (Supplemental Information).

## Electronic supplementary material

Supplementary information

Table S3
