# Supplementary material for: Identification of QTL controlling domestication-related traits in cowpea (Vigna unguiculata L. Walp)
Source: Sci Rep. 2018 Apr 19;8:6261. doi: 10.1038/s41598-018-24349-4 (PMC5908840; doi:10.1038/s41598-018-24349-4)
Supplement: Supplementary file 1 — Supplementary information [file 41598_2018_24349_MOESM1_ESM.docx]

**Identification of QTL controlling domestication-related traits in cowpea (*Vigna unguiculata* L. Walp)**

Sassoum Lo^1^, María Muñoz-Amatriaín^1*^, Ousmane Boukar^2^, Ira Herniter^1^, Ndiaga Cisse^3^, Yi-Ning Guo^1^, Philip A. Roberts^4^, Shizhong Xu^1^, Christian Fatokun^2^ and Timothy J. Close^1^

**Table S1**: Significance level of each trait evaluated in the F8 population

| **Trait** | **Effective degrees of freedom (m0)** | **Critical value** | **Significance level** |
| --- | --- | --- | --- |
| Pod shattering | 4144.08 | 1.21E-05 | 4.92 |
| Peduncle length | 4367.53 | 1.14E-05 | 4.94 |
| Flower color | 1852.01 | 2.70E-05 | 4.57 |
| Days to flowering | 3146.32 | 1.59E-05 | 4.80 |
| 100-seed weight | 4195.80 | 1.19E-05 | 4.92 |
| Pod length | 3881.83 | 1.29E-05 | 4.89 |
| Leaf length | 3946.57 | 1.27E-05 | 4.90 |
| Leaf width | 3666.21 | 1.36E-05 | 4.87 |
| N^o^ seeds per pod | 4738.88 | 1.06E-05 | 4.98 |


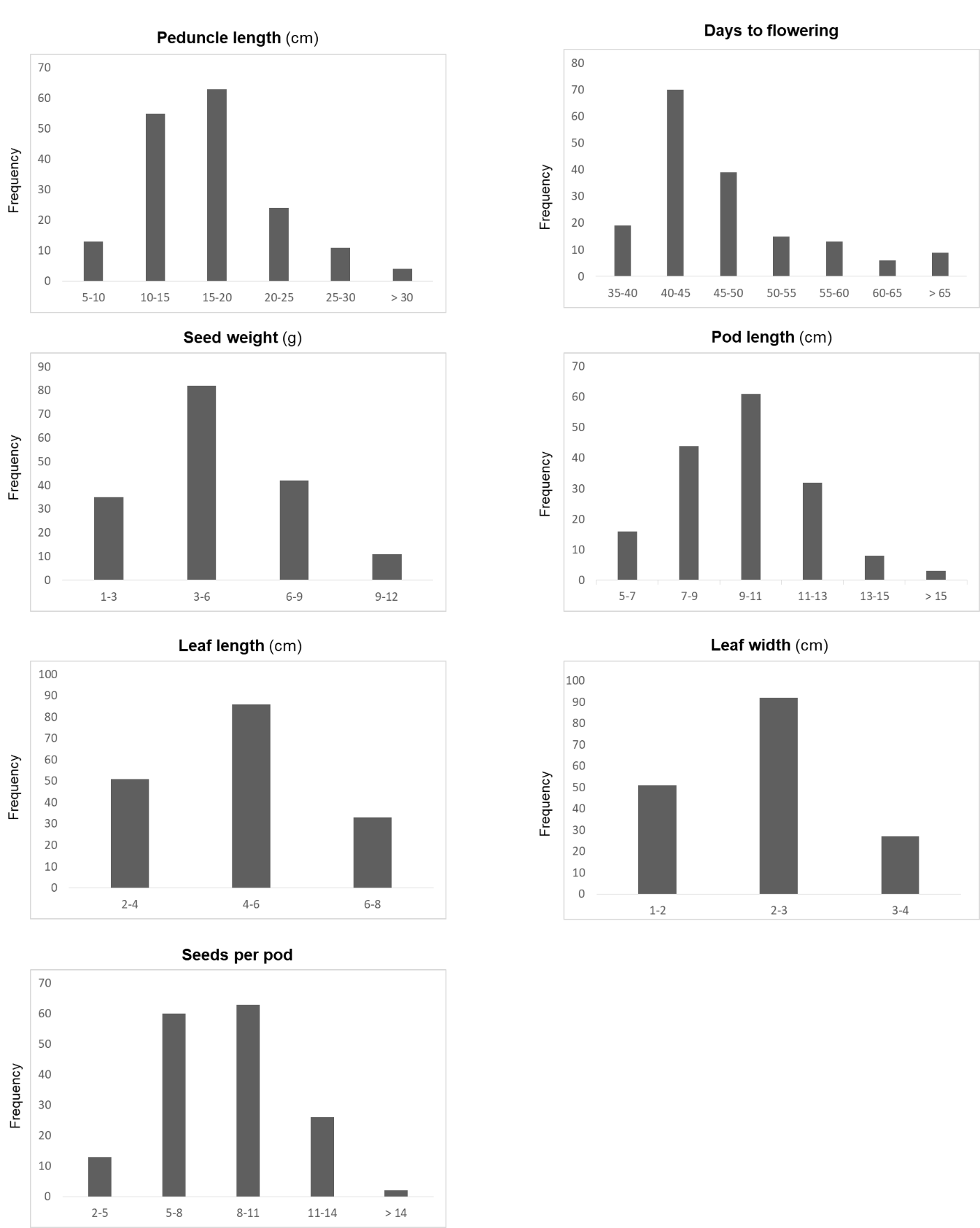


**Fig. S1**: Phenotypic distribution of seven out of the nine domestication-related traits evaluated in the F8 population.
